# Complex PTSD: research directions for nosology/assessment, treatment, and public health

**DOI:** 10.3402/ejpt.v6.27584

**Published:** 2015-05-19

**Authors:** Julian D. Ford

**Affiliations:** Department of Psychiatry, University of Connecticut School of Medicine, Farmington, CT, USA

**Keywords:** PTSD, self-regulation, children, adolescence, assessment, treatment, public health

## Abstract

Complex posttraumatic stress disorder (CPTSD) in children and adolescents extends beyond the core PTSD symptoms to dysregulation in three psychobiological domains: (1) emotion processing, (2) self-organization (including bodily integrity), and (3) relational functioning. CPTSD research directions for the next decade and beyond are identified in three areas: (1) diagnostic classification (establishing the empirical integrity of CPTSD as a distinct form of psychopathology) and psychometric assessment [validation and refinement of measures of childhood polyvictimization and developmental trauma disorder (DTD)], (2) rigorous evaluation and refinement of interventions (and algorithms for their delivery) developed or adapted for CPTSD and DTD, and (3) the epidemiology of CPTSD and DTD, and their public health and safety impact, across the lifespan and intergenerationally, for populations, nations, and cultures.

Children exposed to developmentally adverse interpersonal traumatic stressors (e.g., maltreatment, prolonged family or community violence, torture, exploitation, and genocide)—especially in formative periods (e.g., early childhood and adolescence)—are at risk not only for posttraumatic stress disorder (PTSD) but also for a range of psychiatric disorders (D'Andrea, Ford, Stolbach, Spinazzola, & Van der Kolk, 2012; Ford, 2010). Two decades ago, complex PTSD (CPTSD) was defined as a syndrome involving pathological dissociation, emotion dysregulation, somatization, and altered core schemas of self, relationships, and sustaining beliefs (morality and spirituality) in the aftermath of traumatic victimization (Herman, 1992). Since then dozens of clinical or scientific studies have been conducted on CPTSD (Sar, 2011; Van Dijke et al., 2011), treatment guidelines for adults with CPTSD have been formulated by international professional organizations (Cloitre et al., 2011), and the World Health Organization has included CPTSD as a potential diagnosis in the forthcoming International Classification of Diseases-11th edition (ICD-11) (Cloitre, Garvert, Brewin, Bryant, & Maercker, 2013; Knefel & Lueger-Schuster, 2013).

This article outlines CPTSD research directions for the next decade and beyond in three areas: (1) diagnostic classification (establishing the empirical integrity of CPTSD as a distinct form of psychopathology) and psychometric assessment [validation and refinement of measures of childhood polyvictimization and developmental trauma disorder (DTD)], (2) rigorous evaluation and refinement of interventions (and algorithms for their delivery) developed or adapted for CPTSD, and (3) the epidemiology of CPTSD and DTD and their public health and safety impact, across the lifespan and intergenerationally, for populations, nations, and cultures.

## CPTSD diagnostic classification and psychometric assessment

The construct validity and diagnostic integrity of CPTSD have been challenged as not being grounded in “a clear definition of the disorder, reliable and valid assessment measures, support for convergent and discriminant validity, and incremental validity with respect to implications for treatment planning and outcome” (Resick et al., 2012, p. 241), Yet, based on an exhaustive research review, the PTSD diagnostic criteria in the American Psychological Association's (2013) *DSM-5* were substantially expanded to include symptoms consistent with each CPTSD domain—dysregulation of emotion (i.e., a wide range of persistent negative emotions, self-harm, depersonalization, and derealization), interpersonal functioning (i.e., reckless or aggressive behavior), and self (i.e., persistent negative self-perceptions). Thus, the CPTSD definition proposed for the *ICD-11* is largely incorporated into *DSM-5* PTSD, albeit, with truncated operationalization of affective and interpersonal dysregulation. Research, therefore, is needed to test and refine the operational definition of the CPTSD features both as embedded in and distinct from other PTSD symptoms with adults who have experienced severe childhood traumatization—and to determine the construct validity of CPTSD in relation to personality disorders that involve similar features (Dorrepaal, Thomaes, Smit, et al., 2014; Ford & Courtois, 2014)—both embedded in and apart from the other features of *ICD-11* and *DSM-5* PTSD.

For children and youth, developmental adaptations of CPTSD have been incorporated into a “Developmental Trauma Disorder” syndrome developed by a work group from the US National Child Traumatic Stress Network (www.nctsn.org). The results of an international survey of child-serving clinicians (Ford, Grasso, et al., 2013) indicated that the DTD criteria had clinical utility and were discriminable from childhood internalizing (including PTSD) and externalizing psychiatric diagnoses. In addition to symptom features representing childhood dysregulation in three domains slightly different than those proposed for adult CPTSD (i.e., emotion/physiology, cognition/behavior, and self/relationships), respondents rated a combination of traumatic polyvictimization and attachment disruption as a DTD feature that was highly discriminable from *DSM-*IV diagnoses (including PTSD) and of distinct clinical utility. Therefore, further tests of the construct validity of DTD and its relationship to polyvictimization are a research priority.

Polyvictimization (i.e., exposure to multiple types of interpersonal traumatic stressors) has been identified as a unique risk factor for severe psychosocial problems in childhood that are consistent with DTD (D'Andrea et al., 2012), and cumulative exposure to multiple types of traumatic stressors and re-victimization have been linked to CPTSD in children and adults (Cloitre et al., 2009; Karam et al., 2014). However, varied definitions have been used to operationalize and inform assessments of these constructs representing the burden of traumatic exposure (Grasso, Greene, & Ford, 2013). Research is needed to develop and validate unified definitions and assessments of childhood polyvictimization and cumulative traumatic exposure.

The clinical utility of the proposed DTD symptom set has been tested in a US field trial conducted with 236 parent–child dyads assessed using a new 15-item DTD semi-structured interview (DTD-SI). Study findings (Ford, Spinazzola, van der Kolk, & Grasso, 2014) confirmed DTD-SI inter-rater reliability at the item level. Confirmatory factor analysis showed good fit with a proposed three-criterion structure for DTD (CFI=0.92, RMSEA=0.05, BIC=4395.52), with correlated but distinct and internally consistent (α=0.61–0.72) sub-scales representing emotional, behavioral, and self/relational dysregulation. Construct validity was supported by hierarchical regressions, which showed that, after controlling for *DSM*-IV and *DSM*-5 PTSD, the scores for DTD overall and the three factors were associated with parent-rated measures of dysregulation, alexithymia, and impulse control problems. Additional construct validity evidence was provided by logistic regressions showing that PTSD was uniquely associated with exposure to sexual trauma, emotional abuse, and interpersonal violence, but DTD was uniquely associated with family or community violence and an impaired caregiver. Traumatic loss (separation from a caregiver) was associated with both DTD and PTSD. In terms of comorbidity, the 40% of participating children who met criteria for DSM-IV PTSD (more than half with comorbid DTD) and a comparably large sub-group who met criteria for DTD (also more than half with comorbid PTSD) were two to five times more likely than other participants to meet screening criteria for depressive, manic, psychotic, phobia, separation/generalized anxiety, or obsessive–compulsive disorders. However, DTD, but neither *DSM*-IV nor *DSM*-5 PTSD, was associated with a three- to fourfold increased risk of *DSM*-IV panic, attention deficit hyperactivity disorder (ADHD), oppositional defiant disorder (ODD), conduct disorder (CD), and eating disorders and new or revised *DSM*-5 dysregulation disorders (i.e., non-suicidal self-injury; disruptive social engagement disorder; disruptive mood dysregulation disorder, and reactive attachment disorder).

## Therapeutic interventions for CPTSD

Clinicians in the international survey rated DTD symptoms as only partially remediable by the array of evidence-based interventions for PTSD and other psychiatric disorders (Ford, Grasso et al., 2013). However, there is evidence that traumatically victimized youth (Copping, Warling, Benner, & Woodside, 2001; Dozier, Peloso, Lewis, Laurenceau, & Levine, 2008; Ford, Steinberg, Hawke, Levine, & Zhang, 2012; Harvey & Taylor, 2010; Kagan, 2008; Lowell, Carter, Godoy, Paulicin, & Briggs-Gowan, 2011; Najavits, Gallop, & Weiss, 2006; Spinhoven, Slee, Garnefski, & Arensman, 2009; Connor, Ford, Arnsten, & Greene, 2014) and adults (Dorrepaal, Thomaes, Hoogendoorn, et al., 2014; Ford, Chang, Levine, & Zhang, 2013; Ford, Steinberg, & Zhang, 2011) with DTD/CPTSD clinical features benefit from PTSD treatments delivered with systematic adaptations for these complex forms of dysregulation. Treatment guidelines for adult CPTSD have been developed based on this evidence and ratings by PTSD and CPTSD clinical experts (Cloitre et al., 2011). Research and expert-based treatment guidelines are also needed for children and youth with DTD features.

A three-phase psychotherapy model (i.e., engagement/preparation, trauma processing, and generalization/sustainment) for childhood (Connor et al., 2014) and adult (Cloitre et al., 2011) PTSD and CPTSD/DTD is widely accepted. Although the duration of each phase is not pre-determined (and depending upon the individual client may be accomplished as briefly as in a single session; Courtois & Ford, 2013), the three-phase model has been criticized as unnecessarily delaying the purported “active” treatment phase of trauma processing (Jongh & Broeke, 2014). However, there is evidence that interventions specifically designed to enhance clients' sense of safety, engagement, and efficacy may be associated with treatment completion and outcomes when CPTSD is comorbid with personality disorders (Dorrepaal, Thomaes, Smit, et al., 2014). Also, a meta-analysis of randomized clinical trials with adult PTSD concluded that therapies that do not require trauma memory processing generally have equivalent benefit to trauma memory-processing therapies, particularly with women survivors of sexual abuse/assault (Bisson, Roberts, Andrew, Cooper, & Lewis, 2013). In addition, several promising phase-based interventions for DTD are in development (Ford, Blaustein, Habib, & Kagan, 2013) including one with both randomized clinical trial and quasi-experimental outcome evidence with traumatized youth and adults (Ford, 2015).

Research is needed to adapt the randomized clinical trial design to systematically test varied types, combinations, sequences, lengths, and sub-group matches in order to develop scientifically valid patient-centered algorithms (Almirall, Compton, Gunlicks-Stoessel, Duan, & Murphy, 2012) for PTSD that explicitly address the outcomes for recipients with CPTSD (and DTD). Interventions for DTD children (e.g., refugees) should be built on scientific findings of risk and protective factors. For example, Betancourt et al. (2015) identified “Five forms of resources comprising individual, family, and collective/community strengths: religious faith, healthy family communication, support networks, and peer support.” and noted that “many of these locally occurring protective resources have the potential to be leveraged by family and community-based interventions” (p. 114).

## Epidemiology: the public health and safety impact of CPTSD

Biopsychosocial dysregulation consistent with CPTSD and DTD may contribute to, and place adults and youth at risk not only for a psychopathology but also for physical health problems (Kubzansky et al., 2014; Mason et al., 2014) and diminished access to socio-economic resources (Walter, Hall, & Hobfoll, 2008). When health (Theall, McKasson, Mabile, Dunaway, & Drury, 2013) or social integration (Hall, Chen, Wu, Zhou, & Latkin, 2014; Teng, Hall, & Li, 2014) is impaired, vulnerable individuals and entire populations are at risk for becoming trapped in intergenerational vicious cycles escalating danger, disadvantage, and dysregulation (Olff et al., 2014; Sun et al., 2013; Theall, Brett, Shirtcliff, Dunn, & Drury, 2013). CPTSD is a transcultural phenomenon that occurs worldwide (De Jong, Komproe, Spinazzola, Van der Kolk, & Van Ommeren, 2005). The lifespan and intergenerational public health and safety impact of DTD and CPTSD across populations, nations, and cultures is, therefore, an urgent research agenda.

## Supplementary Material

Complex PTSD: research directions for nosology/assessment, treatment, and public health

Complex PTSD: research directions for nosology/assessment, treatment, and public health

Complex PTSD: research directions for nosology/assessment, treatment, and public health

Complex PTSD: research directions for nosology/assessment, treatment, and public health

Complex PTSD: research directions for nosology/assessment, treatment, and public health

Complex PTSD: research directions for nosology/assessment, treatment, and public health
