# Supplementary material for: Complex PTSD: research directions for nosology/assessment, treatment, and public health
Source: Eur J Psychotraumatol. 2015 May 19;6:10.3402/ejpt.v6.27584. doi: 10.3402/ejpt.v6.27584 (PMC4439420; doi:10.3402/ejpt.v6.27584)
Supplement: Complex PTSD: research directions for nosology/assessment, treatment, and public health [file EJPT-6-27584-s001.pdf]

ESPT complexe dans l'enfance et trouble développemental traumatique: orientations de la recherche pour la nosologie / évaluation, traitement et santé publique

Julian Ford

L'ESPT complexe (ESPTc) chez les enfants et les adolescents s'étend au-delà des principaux symptômes de l'ESPT vers une dérégulation dans trois domaines psychobiologiques: (1) traitement des émotions, (2) auto-organisation (y compris de l'intégrité physique), et (3) fonctionnement relationnel. Les directions de recherche de l'ESPTc pour la prochaine décennie et au-delà sont identifiés dans trois domaines: (a) la classification diagnostic (définition de l'intégrité empirique de l'ESPTc comme une forme distincte de psychopathologie) et des évaluations psychométriques (validation et perfectionnement des mesures de polyvictimisation de l'enfance et le trouble développemental traumatique , DTD) ; (b) l'évaluation et l'amélioration des interventions (et algorithmes pour leur passation) développées ou adaptées pour l'ESPTc et le DTD ; et (c) l'épidémiologie de l'ESPTc et du DTD et de leur impact sur la sécurité et la santé publique, au cours de la vie et de façon intergénérationnelle, pour les populations, nations et cultures.

Mots-clés: ESPT; autorégulation; enfants; adolescence; évaluation; traitement, santé publique

Citation: European Journal of Psychotraumatology 2015, **6**: 27584 - http://dx.doi.org/10.3402/ejpt.v6.27584
