# Supplementary material for: Complex PTSD: research directions for nosology/assessment, treatment, and public health
Source: Eur J Psychotraumatol. 2015 May 19;6:10.3402/ejpt.v6.27584. doi: 10.3402/ejpt.v6.27584 (PMC4439420; doi:10.3402/ejpt.v6.27584)
Supplement: Complex PTSD: research directions for nosology/assessment, treatment, and public health [file EJPT-6-27584-s002.pdf]

Komplexe PTBS bei Kindern und Entwicklungstraumastörung: Forschungsempfehlungen für die Nosologie/Dignostik, Behandlung und die öffentliche Gesundheit

Julian Ford

Komplexe PTBS (KPTBS) bei Kindern und Jugendlichen reicht über die Kernsymptome der PTBS hinaus zur Disregulation in drei psychobiologischen Bereichen: (1) Gefühlsverarbeitung, (2) Selbst-Organisation (eingeschlossen die körperliche Integrität), (3) Funktionieren in Beziehungen. KPTBS Forschungsempfehlungen für zumindest das nächste Jahrzehnt verweisen auf drei Bereiche: (a) diagnostische Klassifikation (Herstellung der empirischen Integrität der KPTBS als spezifische Form der Psychopathologie) und psychometrische Instrumente (Validierung und Verfeinerung der Instrumente für die Messung der Polyviktimisierung bei Kindern und der Entwicklungstraumastörung (DTD), (b) strenge Evaluation und Verfeinerung der Interventionen (und Algorithmen für ihren Einsatz) entwickelt oder adaptiert für KPTSB und DTD, und (c) epidemiologische Forschung für KPTBS und DTD und deren Auswirkungen für die öffentliche Gesundheit und für die Sicherheit, über die Lebensspanne hinweg, ebenso wie über die Generationen, für Bevölkerung, Nationen und Kulturen

Schlüsselwörter. PTBS, Selbstregulation, Kinder, Jugendliche, Diagnostik, Behandlung, öffentliche Gesundheit

Citation: European Journal of Psychotraumatology 2015, **6**: 27584 - http://dx.doi.org/10.3402/ejpt.v6.27584
