# Supplementary material for: Complex PTSD: research directions for nosology/assessment, treatment, and public health
Source: Eur J Psychotraumatol. 2015 May 19;6:10.3402/ejpt.v6.27584. doi: 10.3402/ejpt.v6.27584 (PMC4439420; doi:10.3402/ejpt.v6.27584)
Supplement: Complex PTSD: research directions for nosology/assessment, treatment, and public health [file EJPT-6-27584-s003.pdf]

Gyermekkori komplex PTSD és fejlődési trauma zavar: Kutatási irány a diagnosztika, felmérés, terápia és közegészségügy területein

Julian Ford

A komplex PTSD (CPTSD) gyerekeknél és serdülőknél túlmutat a PTSD vezető tünetein és három pszichobiológiai terület diszregulációját jelenti. Ezek az (1) érzelmi feldolgozás, (2) szelf-szerveződés (beleértve a testi integritást is), és (3) kapcsolati működés. A CPTSD-vel kapcsolatos kutatási irány a következő jó tíz évre három területre osztható fel: (a) diagnosztikus klasszifikáció (a CPTSD, mint különálló zavar empirikus megalapozása) és pszichometrikus vizsgálat (a gyermekkori poliviktimizáció és Fejlődési Trauma Zavar /Developmental Trauma Disorder, DTD/ mérőeszközeinek validálása és pontosítása, (b) a CPTSD és DTD intervencióinak kiértékelése és adaptációja (algoritmusok kialakítása a kezeléshez), (c) a CPTSD és DTD epidemiológiája és társadalomra mért hatása nemzeteken és kultúrákon keresztül.

Kulcsszavak: PTSD; önszabályozás; gyermekek; serdülők; kiértékelés; kezelés; közegészségügy

Citation: European Journal of Psychotraumatology 2015, **6**: 27584 - http://dx.doi.org/10.3402/ejpt.v6.27584
