# Supplementary material for: Complex PTSD: research directions for nosology/assessment, treatment, and public health
Source: Eur J Psychotraumatol. 2015 May 19;6:10.3402/ejpt.v6.27584. doi: 10.3402/ejpt.v6.27584 (PMC4439420; doi:10.3402/ejpt.v6.27584)
Supplement: Complex PTSD: research directions for nosology/assessment, treatment, and public health [file EJPT-6-27584-s004.pdf]

TEPT complejo infantil y trastorno de trauma del desarrollo: líneas de investigación en nosología/evaluación, tratamiento y salud pública:

Julian Ford

El TEPT complejo (TEPTC) en niños y adolescentes se extiende más allá de los síntomas centrales del TEPT hasta la desregulación en 3 áreas psicobiológicas: (1) procesamiento de la emoción, (2) auto organización (incluyendo la integridad corporal) y (3) funcionamiento relacional. Se identifican las líneas de investigación sobre el TEPTC en las próximas de décadas en tres áreas: (a) clasificación diagnóstica (estableciendo la integridad empírica del TEPTC como un tipo diferenciado de psicopatología) y evaluación psicométrica (validación y perfeccionamiento de las mediciones de la polivictimización infantil y el trastorno de trauma del desarrollo, TTD), (b) evaluación rigurosa y perfeccionamiento de las intervenciones (y los algoritmos para poder aplicarlos) desarrolladas o adaptadas para el TEPTC y el TTD y (c) la epidemiología del TEPTC y el TTD y su impacto sobre la salud pública y la seguridad, a lo largo de los ciclos vitales e intergeneracionalmente, para poblaciones, naciones y culturas.

Palabras clave: TEPT; autorregulación; niños; adolescencia; evaluación; tratamiento, salud pública

Citation: European Journal of Psychotraumatology 2015, **6**: 27584 - http://dx.doi.org/10.3402/ejpt.v6.27584
