# Supplementary material for: Complex PTSD: research directions for nosology/assessment, treatment, and public health
Source: Eur J Psychotraumatol. 2015 May 19;6:10.3402/ejpt.v6.27584. doi: 10.3402/ejpt.v6.27584 (PMC4439420; doi:10.3402/ejpt.v6.27584)
Supplement: Complex PTSD: research directions for nosology/assessment, treatment, and public health [file EJPT-6-27584-s005.pdf]

Çocukluk Dönemi Karmaşık TSSB ve Gelişimsel Travma Bozukluğu: Nozoloji/ Değerlendirme, Tedavi ve Kamu Sağlığı için Araştırma Yönergeleri

Julian Ford

Çocuklarda ve ergenlerde Karmaşık TSSB (KTSSB) ana TSSB semptomların ötesinde olup üç psikobiyolojik alandaki düzensizliğe yayılır: 1) duygu işlemleme, 2) özdüzenleme (bedensel bütünlüğü içeren), ve 3) ilişkisel işlevsellik. Gelecek on yıl ve daha sorası için KTSSB araştırma yönergeleri üç alanda tanımlanmıştır: a) tanısal sınıflama (psikopatolojinin farklı bir türü olarak KTSSB'nin deneysel bütünlüğünü kurmak) ve, psikometrik değerlendirme (çocukluk dönemi çoklu mağduriyetin ve Gelişimsel Travma Bozukluğunu, GTB geçerliği ve düzenlemesi), b) KTSSB ve GTB için geliştirilen ya da uyarlanan, müdahalelerin sıkı değerlendirilmesi ve düzenlenmesi (ulaşımı için algoritmaların) ve c) KTSSB ve GTB'nin epidemiyolojisi ve bunların toplumlar, milletler, ve kültürler için toplum sağlığı ve güvenliği üzerindeki etkileri.

Anahtar Kelimeler: TSSB; öz düzenleme; çocuklar; ergenlik; tedavi; toplum sağlığı

Citation: European Journal of Psychotraumatology 2015, **6**: 27584 - http://dx.doi.org/10.3402/ejpt.v6.27584
