# Supplementary material for: Complex PTSD: research directions for nosology/assessment, treatment, and public health
Source: Eur J Psychotraumatol. 2015 May 19;6:10.3402/ejpt.v6.27584. doi: 10.3402/ejpt.v6.27584 (PMC4439420; doi:10.3402/ejpt.v6.27584)
Supplement: Complex PTSD: research directions for nosology/assessment, treatment, and public health [file EJPT-6-27584-s006.pdf]

Kompleksni PTSP u detinjstvu i razvojni traumatski poremećaj: istraživački pravci za nozologiju/procenu, terapiju i javno zdravlje

Julian Ford

Kompleksni PTSP (KPTSP) kod dece i adolescenata se širi mimo ključnih PTSP simptoma ka disregulaciji tri psihobiološka domena: (1) emocionalno procesuiranje, (2) samo-organizacija (uključujući telesni integritet) i (3) interpersonalno funkcionisanje. Pravci KPTSP istraživanja u sledećoj dekadi i kasnije se identifikuju u tri oblasti: (a) dijagnostička klasifikacija (uspostavljanje empirijskog integriteta KPTSP-a kao zasebne forme psihopatologije), i psihometrijska procena (validacija i pročišćavanje merenja dečije poliviktimizacije i razvojnog traumatskog poremećaja, RTP), (b) stroga procena i jasne intervencije (uz algoritme za njihovo sprovođenje) razvijeni ili adaptirani za KPTSP ili RTP i (c) epidemiologija KPTSP-a i RTP-a i njihov uticaj na javno zdravlje i bezbednost, tokom životnog veka i intergenracijski, za populacije, nacije i kulture.

Ključne reči: PTSP; samo-regulacija; deca; adolescent; procena; terapija; javno zdravlje

Citation: European Journal of Psychotraumatology 2015, **6**: 27584 - http://dx.doi.org/10.3402/ejpt.v6.27584
